# Self-assembly and semiconductivity of an oligothiophene supergelator

**DOI:** 10.3762/bjoc.6.122

**Published:** 2010-11-16

**Authors:** Pampa Pratihar, Suhrit Ghosh, Vladimir Stepanenko, Sameer Patwardhan, Ferdinand C Grozema, Laurens D A Siebbeles, Frank Würthner

**Affiliations:** 1 Universität Würzburg, Institut für Organische Chemie, 97074 Würzburg, Germany; 2 Indian Association for the Cultivation of Science, Polymer Science Unit, Kolkata-700032, India (present address); 3 Opto-Electronic Materials Section, Delft Chem Tech, Delft University of Technology, Julianalaan 136, 2628 BL Delft, The Netherlands

**Keywords:** charge transport, hydrogen bonding, oligothiophene, organogel, self-assembly

## Abstract

A bis(trialkoxybenzamide)-functionalized quaterthiophene derivative was synthesized and its self-assembly properties in solution were studied. In non-polar solvents such as cyclohexane, this quaterthiophene π-system formed fibril aggregates with an H-type molecular arrangement due to synergistic effect of hydrogen bonding and π-stacking. The self-assembled fibres were found to gelate numerous organic solvents of diverse polarity. The charge transport ability of such elongated fibres of quaterthiophene π-system was explored by the pulse radiolysis time resolved microwave conductivity (PR-TRMC) technique and moderate mobility values were obtained. Furthermore, initial AFM and UV-vis spectroscopic studies of a mixture of our electron-rich quaterthiophene derivative with the electron acceptor [6,6]-phenyl-C_61_-butyric acid methyl ester (PCBM) revealed a nanoscale segregated assembly of the individual building blocks in the blend.

## Introduction

Self-assembly provides a spontaneous pathway to generate higher-order structures from suitably designed building blocks by virtue of specific intra and intermolecular non-covalent interactions [1]. The development of such building blocks containing various functional π-systems has attracted much interest in the recent past due to their potential applications as active components in a variety of organic electronic devices [2]. Organogels are a special class of self-assembled materials in which small building blocks generate fibrous structures due to intermolecular non-covalent interactions, and these elongated fibres form interpenetrating network in which the solvent molecules are trapped [3–4]. Organogels based on various π-systems such as oligophenylenevinylenes [5] and thienylenevinylenes [6] oligophenyleneethylenes [7], phthalocyanines [8], porphyrins [9], naphthalene and perylene bisimides [10–12], acenes [13–14] and merocyanines [15–16] have been studied in recent years. Self-assembly of various oligothiophene derivatives have been extensively investigated on account of their semiconducting and optoelectronic properties [17]. Feringa and co-workers reported organogels based on bisurea derivatives of bithiophene chromophores and demonstrated a significant charge carrier mobility as a result of self-assembly (Σμ_min_ = 5 × 10^−3^ cm^2^ V^−1^ s^−1^) [18]. However, the building blocks were made only from mono and bithiophene units. Later, Shinkai and co-workers reported the gelation ability of quaterthiophene and hexathiophene derivatives with peripheral amide-linked cholesterol moieties [19]. We recently reported [12] the self-assembly of perylene bisimide (PBI) π-systems that are functionalized with trialkoxybenzamide moiety and have shown that they possess much superior and versatile gelation and self-assembly properties compared to those of cholesterol-linked PBI gelators reported earlier [10]. We also have demonstrated that the trialkoxybenzamide units provide a unique opportunity to tune the mode of self-assembly (H-type vs. J-type) of PBIs by systematically varying the steric crowding in the peripheral alkyl groups [20–21]. These recent findings on PBI gelators prompted us to explore the utility of similar supramolecular design on the self-assembly of other functional π-systems.

**Scheme 1:**
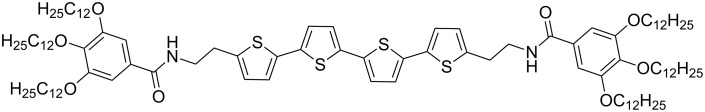
Structure of the quarterthiophene derivative **T1**.

Herein we describe the synthesis, self-assembly, gelation, and charge-carrier mobility studies of trialkoxybenzamide-functionalized quaterthiophene derivative **T1** (Scheme 1). We also present our initial results on the morphology of blends of this electron-rich p-type semiconducting **T1** with the well-known n-type semiconductor [6,6]-phenyl-C_61_-butyric acid methyl ester (PCBM).

## Results and Discussions

### Synthesis

**Scheme 2:**
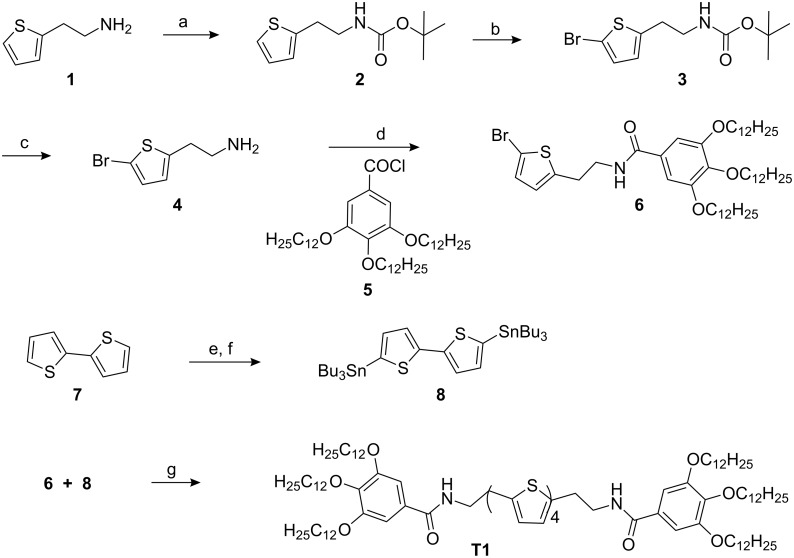
Synthetic route to **T1**. Reagent and conditions: a) Boc anhydride, CH_2_Cl_2_, 6 h, 0 °C–rt, 97%; b) NBS, DMF, rt, 12 h, 89%; c) TFA, CH_2_Cl_2_, rt, 4 h, 99%; d) Et_3_N, CH_2_Cl_2_, 0 °C–rt, 78%; e) BuLi, THF, 0 °C, 1 h; f) Bu_3_SnCl, rt, 12 h; g) Pd(PPh_3_)_2_Cl_2_, DMF, 80 °C, 8 h, 82%.

The synthetic route to the newly designed quaterthiophene derivative **T1** is depicted in Scheme 2. Compounds **4** [22] and **5** [20] were synthesized from commercially available starting materials in a few synthetic steps by literature methods, and then coupled together to produce the thiophene-containing building block **6** in 78% yield. 2, 2´-Bithiophene was converted to the corresponding tributyltin derivative **8** by the reported procedure [19] and then coupled with compound **6** in the presence of a Pd-catalyst to give the desired oligothiophene derivative **T1** in 82% yield. The new compounds **6** and **T1** were characterized by ^1^H NMR and UV-vis spectroscopy, HRMS (ESI) and elemental analysis, while those synthetic intermediates already reported in the literature were characterized by ^1^H NMR and UV-vis spectroscopy.

### Gelation tests

**Figure 1:**
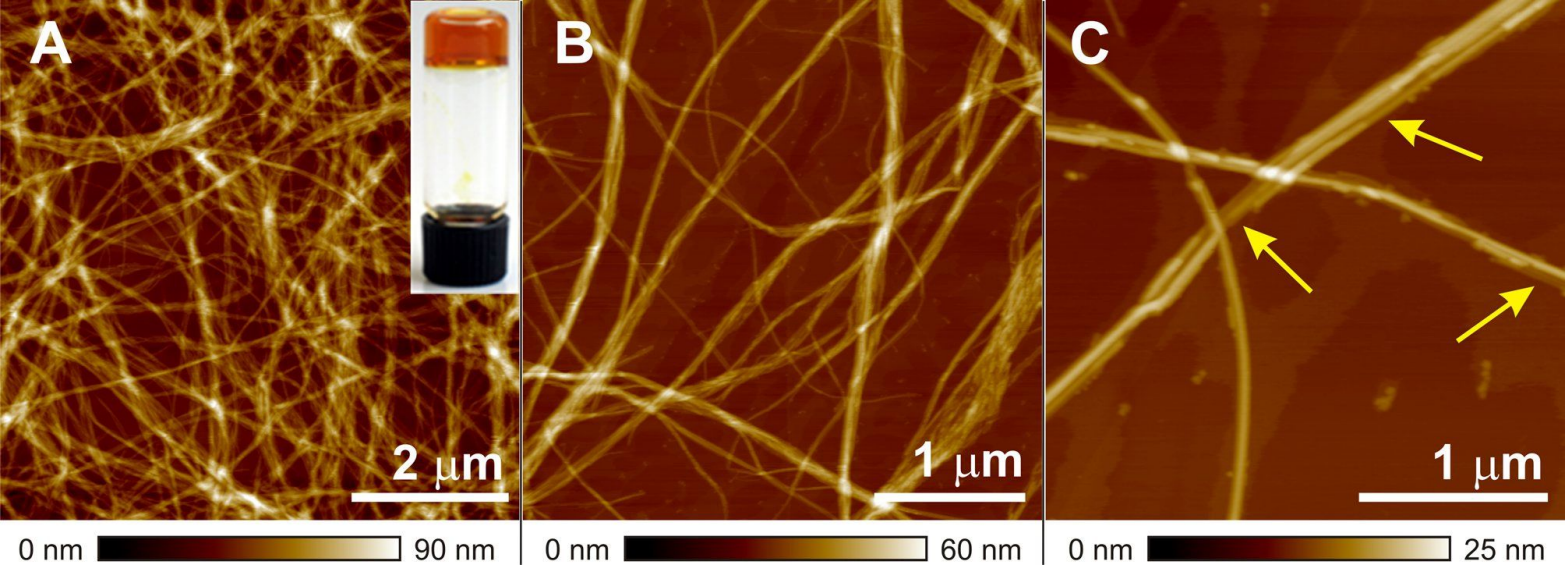
AFM height images of a film spin-coated from diluted gel solution of **T1** in MCH (2 × 10^−3^ M) onto HOPG (A, B and C). The z scale (90, 60 and 25 nm, respectively) is shown under the respective image. Inset in 1A: a photograph of gel in cyclohexane at 1.5 mM concentration.

The gelation properties of **T1** were examined in various organic solvents (Table 1) at a concentration of 3.5 mM. At room temperature **T1** was soluble only in chloroform, dichloromethane, and tetrahydrofuran among other tested solvents. However, at elevated temperatures this quaterthiophene could be dissolved in all the tested solvents and when the hot solution was cooled down to room temperature, spontaneous gelation was observed (see Figure 1A, inset) in most cases within 5–10 minutes. For example, aromatic hydrocarbons (toluene, benzene), aliphatic hydrocarbons (methylcyclohexane, cyclohexane, *n*-heptane, *n*-hexane), chlorinated hydrocarbons (chlorobenzene, tetrachloroethylene, 1,2-dichloroethane), ethers (dibutyl ether, THF, dioxan), hydrogen-bonding donor molecules (triethylamine, acetone), and even highly polar solvents such as DMF and ethanol could be gelated with **T1**. The only exceptions were acetonitrile and DMSO for which no gelation was observed. Critical gelation concentrations (CGC) were determined for all the solvents gelated with **T1** and found to be below 0.3 wt %. This CGC value is much lower compared to those of the previously reported quaterthiophene gelators containing cholesteryl amide peripheral groups [19]. It is interesting to note that in aliphatic hydrocarbons (methylcyclohexane (MCH), cyclohexane, *n*-hexane, *n*-heptane) and in tetrachloroethylene the CGC values are even less than 0.1 wt %. Organogelators with such low CGC values are classified as supergelators [4], and thus the present quaterthiophene gelator **T1** belongs to this category. The ability of **T1** to gelate such a wide range of solvents and its significantly low CGC values can be attributed to the favourable balance between the self-assembly propensity of the gelator and good solubility due to the presence of the trialkoxybenzamide groups.

**Table 1:** Gelation studies of **T1** in various solvents.

Entry	Solvent	Observation	CGC (moles/lit.)	CGC (Wt %)

1	Toluene	G	1 × 10^−3^	0.20
2	Benzene	G	1 × 10^−3^	0.19
3	Methylcyclohexane	G	2 × 10^−4^	**0.04**
4	Cyclohexane	G	4 × 10^−4^	**0.08**
5	*n*-Heptane	G	3.75 × 10^−4^	**0.09**
6	*n*-Hexane	G	0.4 × 10^−3^	**0.10**
7	CHCl_3_	soluble	**_**	**_**
8	CH_2_Cl_2_	soluble	**_**	**_**
9	Chlorobenzene	G	0.33 × 10^−2^	0.51
10	Tetrachloroethylene	G	9.09 × 10^−4^	**0.09**
11	1,2-Dichloroethylene	G	1.25 × 10^−3^	0.17
12	1,4-Dioxan	G^a^	0.125 × 10^−2^	0.26
13	THF	soluble	**_**	**_**
14	Dibutylether	G	1 × 10^−3^	0.22
15	Acetone	G^a^	1.11 × 10^−3^	0.24
16	Triethylamine	G	0.5 × 10^−3^	0.12
17	Acetonitrile	not soluble	**_**	**_**
18	DMF	G	1 × 10^−3^	0.18
19	DMSO	gel-like ppt.	**_**	**_**
20	Ethanol	G^a^	1 × 10^−3^	0.21

^a^Opaque gel.

### AFM investigations

The topology of gels of quaterthiophene derivative **T1** was examined by atomic force microscopy (AFM) in the tapping mode. As an illustrative example, the AFM images of **T1** gel in MCH deposited on highly ordered pyrolytic graphite (HOPG) are shown in Figure 1. These images clearly show interconnected long fibers a few micrometers in length and bundles that are responsible for the gelation of the solvents. At nanometer to micrometer resolution, a fibrous network is observed that contains smaller fibers (indicated by yellow arrows in Figure 1C) with a mean height of 2.3±0.3 nm and width of 7.0±1.0 nm.

### Self-assembly studies by UV-vis spectroscopy

**Figure 2:**
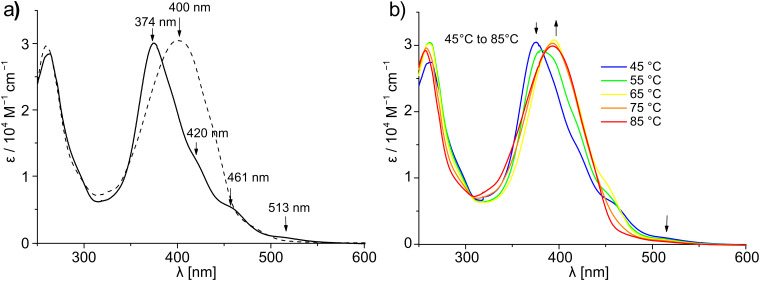
a) UV-vis spectra of **T1** in chloroform (dashed line) and *n*-heptane (solid line); b) UV-vis spectra of **T1** in *n*-heptane at different temperatures. Concentration of **T1** is 5 x 10^−5^ M.

Self-assembly of **T1** in solution was examined by UV-vis spectroscopy. Chloroform is known to be an excellent solvent for the study of rigid π-systems [20–21], and the oligothiophene chromophore **T1** has good solubility in this solvent. However, as noted above, in nonpolar solvents such as *n*-heptane, gelation was observed at extremely low CGC values. Thus, we compared the UV-vis spectra of **T1** in chloroform and *n*-heptane at 0.05 mM concentration (Figure 2a).

**Scheme 3:**
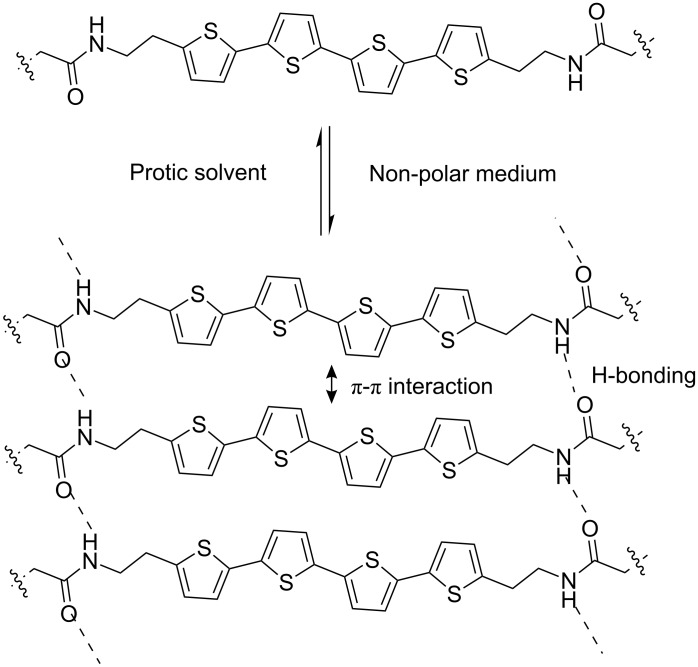
Proposed mode of self-assembly of **T1**.

**Figure 3:**
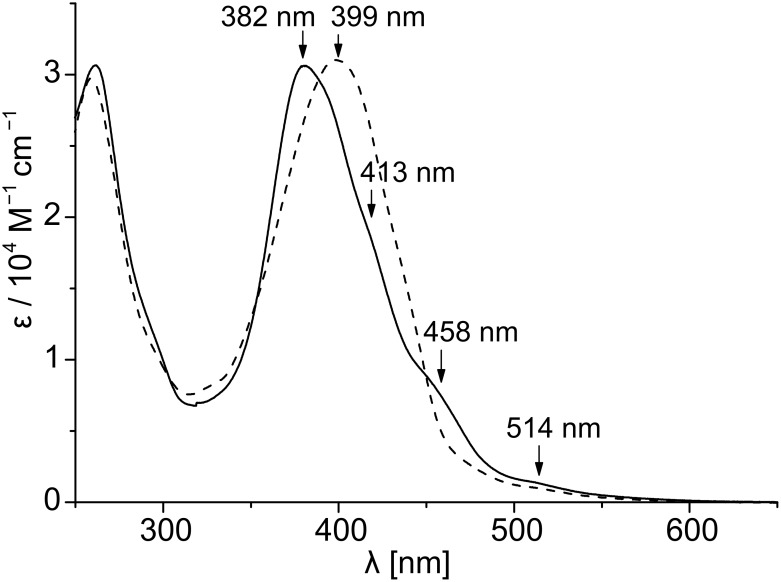
UV-vis spectra of **T1** (concentration 5 x 10^−5^ M) in cyclohexane (solid line) and 2.4% MeOH in cyclohexane (dashed line) at 25 °C.

In chloroform the π-π* transition band appeared at 400 nm, whilst in *n*-heptane it was shifted significantly to a lower wavelength (374 nm). In *n*-heptane, additional shoulders appeared at 420, 461 and 513 nm that were absent in the spectrum recorded in chloroform. These results are in accord with the literature reports for the formation of H-aggregates of oligothiophenes [19] and can be attributed to an excitonic interaction as well as an increase in the conformational order in the assembled state [23]. To test the reversibility of the self-assembly process, variable temperature UV-vis experiments were performed in *n*-heptane. These clearly showed that when the temperature was gradually raised from 45 °C to 85 °C, the spectral pattern changed significantly (Figure 2b). The λ_max_ value shifted from 374 nm to 400 nm and the shoulders at higher wavelengths (420, 461 and 513 nm) gradually disappeared. At the higher temperature, the spectrum resembles quite well that observed in chloroform solution (Figure 2a). These results suggest disassembly of the H-type aggregate to monomeric building blocks at elevated temperature. The self-assembly of **T1** at such low concentrations can be attributed to the synergistic effect of π-π-stacking among the oligothiophene chromophores and intermolecular hydrogen bonding between the amide groups of the neighbouring chromophores (Scheme 3). To ascertain the involvement of hydrogen bonding in the self-assembly, we examined the effect of a protic solvent, e.g. MeOH, on the self-assembly process by UV-vis spectroscopy. MeOH itself can be involved in H-bonding interaction with the amide groups, and thus expected to interfere with the inter-chromophoric hydrogen-bonding interaction and to disrupt the assembly. Note that, as methanol is not miscible with *n*-heptane, cyclohexane was used as nonpolar solvent for this experiment. It can be seen in Figure 3 that in the presence of 2.4% MeOH the absorption spectrum of **T1** in cyclohexane changed significantly. The shoulders at longer wavelength disappeared and the λ_max_ value shifted from 382 nm to 399 nm, suggesting disassembly of the aggregate into monomers. Thus, it is evident that hydrogen bonding is essential for the self-assembly of **T1**, particularly at such a low concentration of 5 x 10^−5^ M.

### Studies on blends of T1 with PCBM

**Figure 4:**
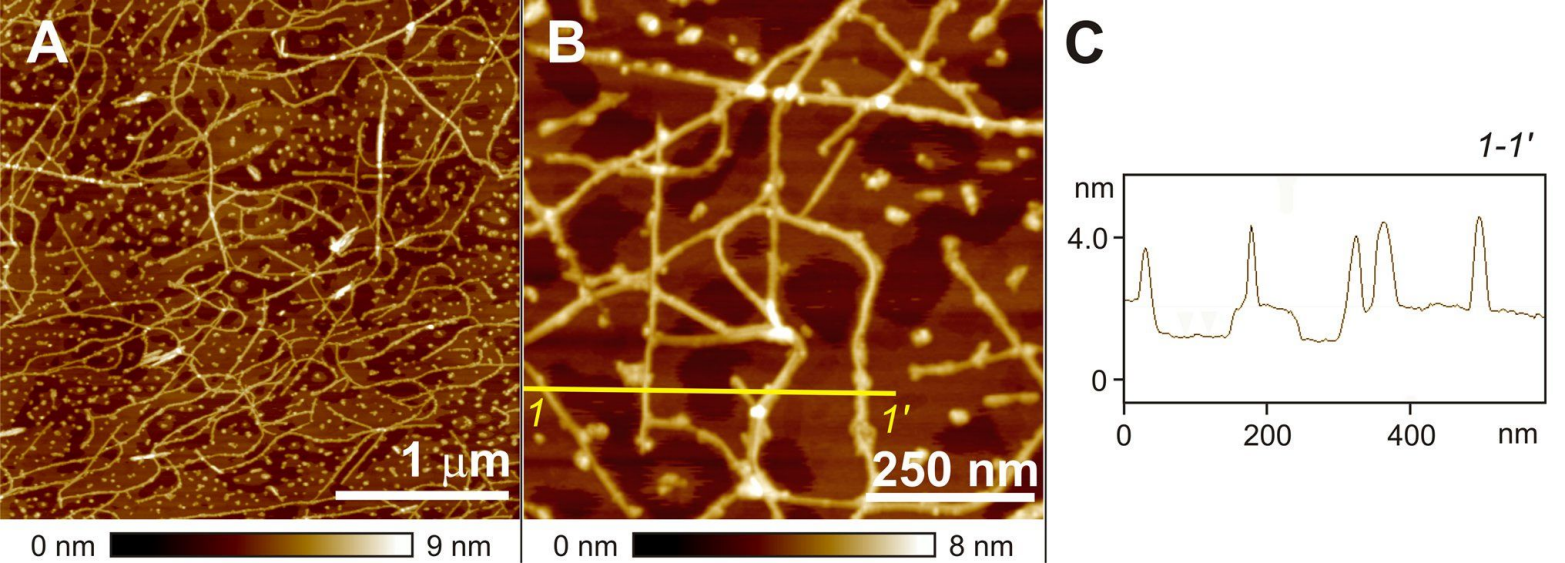
AFM height images (A and B) of a film spin-coated from MCH solution (concentration 5 x 10^−4^ M) of a 1:1 mixture of **T1** and PCBM onto HOPG. The z scale (9 nm and 8 nm, respectively) is shown under the respective image. Figure 4C depicts cross-section analysis along the yellow line 1-1' in image B.

The fullerene derivative [6,6]-phenyl-C_61_-butyric acid methyl ester (PCBM) is a well-known n-type semiconductor and its blends with various electron-donor materials have been extensively used in solar cell devices [24]. The morphology of the blend of donor and acceptor materials plays a prominent role in device performance [25]. Recently, we reported that a n-type perylene bisimide organogelator exhibits photovoltaic activity with a p-type semiconducting polymer [26]. In this work, as an initial study we characterized the morphology of a mixture of PCBM with the p-type semiconducting oligothiophene-based gelator **T1** to elucidate the prospect of this system being used as a photovoltaic material. Figure 4 depicts the AFM images of a mixture of **T1** and PCBM on HOPG in which two types of aggregates are visible. The observed long fibers can be attributed to aggregates of **T1** and the spherical nano-objects can be related to PCBM aggregates. The height and width of the long fibers were estimated to be 2.4±0.3 nm and 6.0±01.0 nm, respectively, which very closely match with the values observed for the self-assembly of **T1** alone (Figure 1). This indicates that PCBM does not interfere in the self-assembly of **T1**.

### Determination of charge carrier mobility

It was anticipated that the well-organized π-stacked assembly of oligothiophene chromophores would provide percolation pathways for charge transport, similar to the previously reported results for mono- and bithiophene bisurea compunds [18]. Therefore, the charge transport properties of **T1** in the solid state were investigated by pulse radiolysis time resolved microwave conductivity (PR-TRMC) measurements [27].

**Figure 5:**
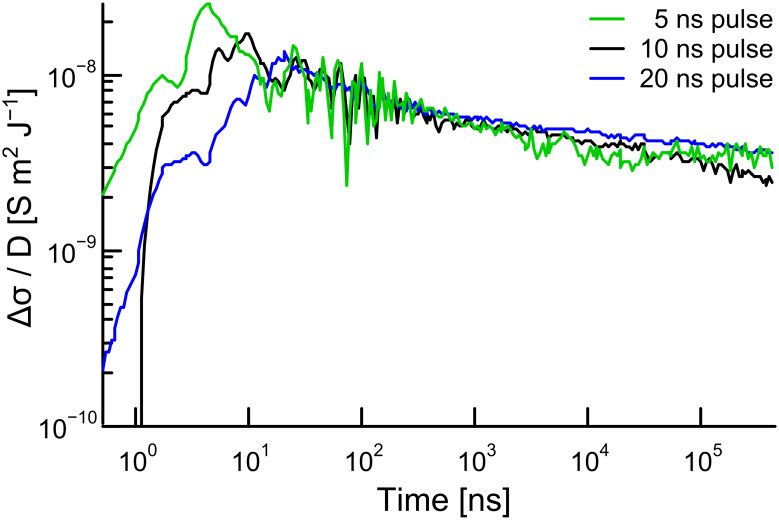
Variation of dose-normalized conductivity transients (Δσ/*D*) with time for **T1**.

PR-TRMC measurements were performed with a solid (powder) sample of **T1** using the same methodology as reported previously [27–28]. The sample (~32.5 mg) was irradiated with a short pulse of 3 MeV electrons, which lead to the formation of charges in the material during the pulse. The conductivity was measured as a function of time by microwave conductivity measurements. The dose-normalized PR-TRMC transients for different irradiation doses at a temperature of 20 ºC are shown in Figure 5. The decay is the same for all irradiation doses, which indicates first order decay of the charge carriers. Such first order decay is typically observed for columnar materials and can be due to either trapping of charges or geminate recombination of electrons and holes [28].

The lower limits of Σμ_min_ (the sum of the mobilities of the positive and negative charge carriers) were estimated from the relation Σμ_min_ = *E_p_*(Δσ/*D*), where *E_p_* is the average electron-hole pair formation energy [28]. The mobility values obtained in this way at various temperatures are listed in Table 2. The charge carrier mobility of **T1** at 20 °C was found to be 2.9 × 10^−3^ cm^2^ V^−1^ s^−1^. When the sample was heated up to 110 °C, no significant difference was observed in the mobility value compared to that at 20 °C, indicating good thermal stability of the supramolecular assembly of **T1**. A repeat measurement of mobility at 20 °C after the sample was annealed revealed a slightly higher value (4.2 × 10^−3^ cm^2^ V^−1^ s^−1^), indicating an improvement of the supramolecular order in the material on annealing. It is interesting to note that the mobility value obtained for **T1** is very close to that reported for non-regio regular alkyl-substituted polythiophenes (Σμ_min_ = 7 × 10^−3^ cm^2^ V^−1^ s^−1^) [29], suggesting similar electronic coupling between oligothiophenes in the gelated π-stacks.

**Table 2:** Charge carrier mobilities of **T1** at various temperatures in the sequence as measured in two experimental series.

T (°C)	Σμ_min_ (cm^2^ V^−1^ s^−1^)	T (°C)	Σμ_min_ (cm^2^ V^−1^ s^−1^)

20	0.0030	80	0.0024
0	0.0032	100	0.0021
−20	0.0031	110	0.0024
−40	0.0030	100	0.0027
−20	0.0030	80	0.0031
0	0.0032	60	0.0034
20	0.0029	40	0.0039
40	0.0028	20	0.0042
60	0.0026		

## Conclusion

We have demonstrated the self-assembly and charge transport properties of a trialkoxybenzamide-functionalized quaterthiophene π-system. Our studies revealed the versatile and very effective gelation ability of the present system compared to the previously reported oligothiophene gelators. The critical gelation concentrations in numerous solvents are remarkably low and in few cases even in the range of supergelators. The impact of facile self-assembly of newly developed oligothiophene building block on its material properties is reflected in promising charge transport characteristics as revealed by PR-TRMC measurements. When compared with ordinary amorphous or crystalline organic semiconductors it should be taken into account that the oligothiophene moiety, which is responsible for charge transport, is only 19% of the total molecular weight of **T1** and the remainder being made up by long alkyl chains that do not contribute to charge carrier mobility. AFM studies revealed self-sorted assembly of p-type oligothiophene gelator and n-type **PCBM** in their blend which might offer some prospect for photocurrent generation [30].

## Experimental

**Materials and methods.** All reagents and solvents were purchased from commercial sources and purified by standard protocols [31]. Spectroscopic grade solvents were used as received for spectroscopic studies. ^1^H NMR spectra were recorded on a 400 MHz Bruker NMR spectrometer with tetramethylsilane (TMS) as the internal standard. UV-vis experiments were carried out on a Perkin-Elmer Lambda 40P spectrometer equipped with a Peltier system for temperature control.

**Gelation tests.** A measured amount of **T1** and the appropriate solvent were taken together in a sample vial, heated to dissolve the sample and then left at room temperature. After 30 minutes, the gelation was tested by the “stable to inversion” method.

**UV-vis spectroscopic studies.** A stock solution of **T1** was prepared in chloroform with 1 mM concentration. A measured amount of stock solution was transferred to another vial and the solvent removed by blowing argon gas over it. To this vial, a measured amount of solvent such as *n*-heptane or cyclohexane was added to give the desired concentration and the mixture then gently heated in a warm water-bath to yield a homogeneous solution. The solutions were allowed to equilibrate at room temperature for 2 h before performing UV-vis experiments. For variable temperature studies, the temperature was changed from lower to higher values and the sample equilibrated for 10 min prior to each measurement after the particular temperature was reached.

**Atomic force microscopy (AFM) measurements.** AFM measurements were carried out under ambient conditions with a Veeco MultiMode^TM^ Nanoscope IV system operating in the tapping mode in air. Silicon cantilevers (Olympus Corporation, Japan) with a resonance frequency of ~300 kHz and spring constant of ~42 N/m were used. A solution of **T1** in methylcyclohexane (MCH) with a concentration of 2 × 10^−3^ M was spin-coated onto highly oriented pyrolytic graphite (HOPG) at 6000 rpm. The sample of a **T1**-PCBM mixture was prepared by spin-coating of MCH solution with a concentration of 5 × 10^−4^ M onto HOPG at 4000 rpm. The height of the observed fibers was determined by statistical analysis on the premise that the fibers lay on a thin film. Note that, due to the AFM tip broadening effect, the actual width of aggregates is usually smaller than the apparent one.

**Pulse radiolysis time resolved microwave conductivity (PR-TRMC) measurements.** Conductivity measurements of quaterthiophene **T1** were performed on a solid (powder) sample (about 32.5 mg) that was manually compressed into a perspex container. The sample was placed in a microwave cell, consisting of a piece of rectangular waveguide with inner dimensions of 3.55 × 7.00 mm^2^ and short circuited with a metal end plate. The materials were uniformly ionized with a nanosecond pulse of 3 MeV electrons from a Van de Graaff accelerator. The energy absorbed by the sample is accurately known from dosimetry and leads to the formation of a micromolar concentration of charge carriers (ca. 10^−21^ m^−3^). The change in conductivity due to creation of these charges was measured by time resolved microwave conductivity (TRMC) measurements [27].

**Synthesis and characterization.** 3,4,5-Tris(dodecyloxy)benzoyl chloride (**5**) and compounds **2**, **3**, **4** and **8** were prepared according to literature reported procedures and characterized by ^1^H NMR and UV-vis spectroscopy. New compounds (**6** and **T1**) were characterized by ^1^H NMR, UV-vis, HRMS (ESI) and elemental analysis.

***Tert*-butyl 2-(thiophen-2-yl)ethylcarbamate (2).** To a solution of 2-(thiophen-2-yl) ethylamine (1.63 g, 0.013 mol) in 10 mL CHCl_3_, a solution of Boc-anhydride (2.8 g, 0.013 mol) in 10 mL CHCl_3_ was added dropwise and the reaction mixture stirred under an inert atmosphere for 6 h at room temperature. The volatiles were then removed at reduced pressure to yield the crude **2** as a colourless oil (97%). ^1^H NMR (400 MHz, CDCl_3_, TMS, 300 K): *δ* (ppm) = 7.15 (d, 1H), 6.95–6.83 (m, 1H), 6.83 (m, 1H), 4.65 (broad peak, 1H), 3.38 (t, *J* = 6.24 Hz, 2H), 3.01(t, *J* = 6.68 Hz, 2H), 1.44 (s, 9H); UV-vis (CH_2_Cl_2_): λ_max_ (*ε*) = 235 nm (0.697 × 10^4^ M^−1^cm^−1^).

***Tert*-butyl 2-(5-bromothiophen-2-yl)ethylcarbamate (3).** To an ice-cold solution of compound **2** (0.77 g, 3.38 mmol) in 10 mL DMF, a solution of NBS (0.603 g, 3.38 mmol) in 5 mL DMF was added dropwise. After the addition was complete, the reaction mixture was stirred at room temperature for a further 12 h, then poured into 100 mL water and extracted with (2 × 30) mL diethylether. The combined organic layer was dried over anhydrous Na_2_SO_4_ and solvent removed to give the crude product as a light brown oil (90%) which was used in the next step without further purification. ^1^H NMR (400 MHz, CDCl_3_, TMS, 300 K): *δ* (ppm) = 6.88 (d, *J* = 3.64 Hz, 1H), 6.59 (d, *J* = 3.72 Hz, 1H), 4.63 (broad peak, 1H), 3.35 (t, *J* = 5.44 Hz, 2H), 2.93 (t, *J* = 6.60 Hz, 2H), 1.44 (s, 9H); UV-vis (CH_2_Cl_2_): λ_max_ (*ε*) = 238 nm (0.735 × 10^4^ M^−1^cm^−1^).

**2-(5-Bromothiophen-2-yl)ethylamine (4).** To a solution of *tert*-butyl 2-(5-bromothiophene-2-yl)ethylcarbamate (**3**) in 5 mL CH_2_Cl_2_, 5 mL TFA was added and the reaction mixture stirred at rt under an argon atmosphere for 2 h. The volatiles were then removed under reduced pressure to afford the crude product as a light brown oil (96%) which was used in the next step without further purification. ^1^H NMR (400 MHz, CDCl_3_, TMS, 300 K): *δ* (ppm) = 7.48 (broad s, 2H); 6.90 (d, *J* = 3.72 Hz, 1H), 6.67 (d, *J* = 3.64 Hz, 1H), 3.28 (m, 2H), 3.15 (t, *J* = 7.04 Hz, 2H); UV-vis (CH_2_Cl_2_): λ_max_ (*ε*) = 238 nm (0.741 × 10^4^ M^−1^cm^−1^).

***N*-2-(5-Bromothiophen-2-yl)ethyl) 3,4,5-tris(dodecyloxy)benzamide (6).** Compound **4** (2.94 mmol) was dissolved in 5 mL dry CH_2_Cl_2_ and cooled in an ice-bath. To this cold solution, 4 mL triethylamine was added slowly. The resulting mixture was ice-cooled for an additional 10 minutes and then a solution of compound **5** in 10 mL dry CH_2_Cl_2_ was added dropwise. The reaction mixture was stirred at rt for 12 h, diluted with a further 25 mL CH_2_Cl_2_ and washed successively with water (2 × 50 mL), dil. HCl (2 × 50 mL) and finally with 50 mL brine. The combined organic layer was dried over anhydrous Na_2_SO_4_ and solvent removed under reduced pressure to give the crude product as a light yellow solid which was purified by column chromatography on silica gel with CH_2_Cl_2_ as eluent. The product was further purified by dissolving it in 5 mL CH_2_Cl_2_ and re-precipitating from 200 mL *n*-hexane to yield a white solid (67%). M.p. 76–78 °C; ^1^H NMR (400 MHz, CDCl_3_, TMS, 300 K): *δ* (ppm) = 6.91–6.90 (m, 3H), 6.64 (d, *J* = 3.64 Hz, 1H), 6.13 (broad s, 1H), 4.00–3.96 (m, 6H), 3.69–3.63 (m, 2H), 3.07 (t, *J* = 6.44 Hz, 2H), 1.76–1.48 (m, 60H), 0.88 (t, *J* = 7.00 Hz, 9H); UV-vis (CH_2_Cl_2_): λ_max_ (*ε*) = 257 nm (1.381 × 10^4^ M^−1^ cm^−1^), 290 nm (0.296 × 10^4^ M^−1^ cm^−1^); HRMS (ESI): m/z calcd for C_49_H_85_BrNO_4_S [*M* + 2H]^+^: 862.5372; found: 862.5377; elemental analysis: calcd for C_49_H_85_BrNO_4_S: C, 68.18, H, 9.81, N, 1.62, found: C, 67.93, H, 9.70, N, 1.76.

**Quaterthiophene gelator T1.** *n*-BuLi (360 μL in 2 mL dry THF) was added to a round-bottomed flask, flushed with argon gas for 15 minutes, and then cooled to 0 °C in an ice-bath. To this solution, a solution of 2, 2´-bithiophene in dry THF (68.0 mg in 5 mL) was added dropwise under continuous flow of argon. A white solid precipitate was formed. The reaction mixture was stirred at room temperature for 1 h and then immersed in an ice-bath. To this cold solution, 400 μL Bu_3_SnCl was added which caused the precipitate to dissolve immediately. The reaction mixture was stirred at room for further 12 h under an argon atmosphere. The volatiles were removed under reduced pressure to give the crude product as a white pasty mass. The crude product was dissolved in 20 mL dry DMF, compound **9** added and the flask evacuated, purged three times with argon and ~15 mg of the Pd-catalyst added under continuous flow of argon. The reaction mixture was then heated at 80 °C for 8 h under an argon atmosphere. It was observed that an orange precipitate appeared within first 30 min, which almost dissolved as the reaction progressed. After 8 h the reaction was stopped, cooled to rt and poured into 200 mL MeOH. A yellowish orange precipitate was separated by filtration and dried in vacuum to give the crude product as a yellow solid. The crude product was purified by column chromatography on silica gel with 2% methanol in chloroform as eluent to afford the pure product as a yellow solid (78%). M.p. 144 °C. ^1^H NMR (400 MHz, CDCl_3_, TMS, 300 K): δ (ppm) = 7.04–7.00 (m, 6H), 6.79 (s, 4H), 6.18 (s, 2H), 6.78–6.75 (m, 2H), 3.99–3.95 (m, 12H), 3.73–3.69 (m, 4H), 3.14–3.10 (m, 4H),1.81–1.25 (m, 120H), 0.89–0.85 (m, 18H); UV-vis (CH_2_Cl_2_): λ_max_ (*ε*) = 262 nm (1.76 × 10^4^ M^−1^ cm^−1^), 405 nm (2.90 × 10^4^ M^−1^ cm^−1^); HRMS (ESI): m/z calcd for C_106_H_172_N_2_Na_1_O_8_S_4_ [*M* + Na]^+^ :1752.1881; found: 1752.1920; MS (MALDI) m/z calcd for C_106_H_172_N_2_O_8_S_4_ [M + H]^+^ 1730.206, found: 1730.265; elemental analysis: calcd for C_106_H_172_N_2_Na_1_O_8_S_4_: C, 73.56, H, 10.02, N, 1.62, found: C, 73.32, H, 9.81, N, 1.73.

## References

[1] Lehn J-M (1995). Supramolecular Chemistry - Concepts and Perspectives.

[2] Hoeben F M, Jonkheijm P, Meijer E W, Schenning A P H J (2005). Chem Rev.

[3] Fages F (2005). Low Molecular Mass Gelators.

[4] Bouas-Laurent H, Desvergne J-P, Weiss G R, Terech P (2006). Molecular Gels: Materials with Self-Assembled Fibrilla Networks.

[5] Ajayaghosh A, Praveen V K (2007). Acc Chem Res.

[6] Prasanthkumar S, Saeki A, Seki S, Ajayaghosh A (2010). J Am Chem Soc.

[7] Ajayaghosh A, Varghese R, Mahesh S, Praveen V K (2006). Angew Chem, Int Ed.

[8] Engelkamp H, Middelbeek S, Nolte R J M (1999). Science.

[9] Malik S, Kawano S-i, Fujita N, Shinkai S (2007). Tetrahedron.

[10] Sugiyasu K, Fujita N, Shinkai S (2004). Angew Chem, Int Ed.

[11] Mukhopadhyay P, Iwashita Y, Shirakawa M, Kawano S-i, Fujita N, Shinkai S (2006). Angew Chem, Int Ed.

[12] Li X-Q, Stepanenko V, Chen Z, Prins P, Siebbeles L D A, Würthner F (2006). Chem Commun.

[13] Del Guerzo A, Olive A G L, Reichwagen J, Hopf H, Desvergne J-P (2005). J Am Chem Soc.

[14] Desvergne J P, Olive A G L, Sangeetha N M, Reichwagen J, Hopf H, Del Guerzo A (2006). Pure Appl Chem.

[15] Yagai S, Ishii M, Karatsu T, Kitamura A (2007). Angew Chem, Int Ed.

[16] Yao S, Beginn U, Greß T, Lysetska M, Würthner F (2004). J Am Chem Soc.

[17] Mishra A, Ma C-Q, Bäuerle P (2009). Chem Rev.

[18] Schoonbeek F S, Van Esch J H, Wegewijs B, Rep D B A, De Haas M P, Klapwijk T M, Kellogg R M, Feringa B L (1999). Angew Chem, Int Ed.

[19] Kawano S, Fujita N, Shinkai S (2005). Chem–Eur J.

[20] Ghosh S, Li X-Q, Stepanenko V, Würthner F (2008). Chem–Eur J.

[21] Würthner F, Bauer C, Stepanenko V, Yagai S (2008). Adv Mater.

[22] Venkatachalam T K, Sudbeck E A, Uckun F M (2001). Tetrahedron Lett.

[23] Langeveld-Voss B M W, Waterval R J M, Janssen R A J, Meijer E W (1999). Macromolecules.

[24] Nelson J (2002). Curr Opin Solid State Mater Sci.

[25] Van Duren J K J, Yang X, Loos J, Bulle-Lieuwma C W T, Sieval A B, Hummelen J C, Janssen R A J (2004). Adv Funct Mater.

[26] Wicklein A, Ghosh S, Sommer M, Würthner F, Thelakkat M (2009). ACS Nano.

[27] Warman J M, de Haas M P, Dicker G, Grozema F C, Piris J, Debije M G (2004). Chem Mater.

[28] Warman J M, Van de Craats A M (2003). Mol Cryst Liq Cryst.

[29] Van der Laan G P, Haas M P D, Buik A, De Ruiter B (1993). Synth Met.

[30] Yamamoto Y, Fukushima T, Suna Y, Ishii N, Saeki A, Seki S, Tagawa S, Taniguchi M, Kawai T, Aida T (2006). Science.

[31] Perrin D D, Armarego W L F, Perrin D R (1980). Purification of Laboratory chemicals.

